# Bicarbonate Therapy for Critically Ill Patients with Metabolic Acidosis: A Systematic Review

**DOI:** 10.7759/cureus.4297

**Published:** 2019-03-22

**Authors:** Sanniya Khan Ghauri, Arslaan Javaeed, Khawaja Junaid Mustafa, Anna Podlasek, Abdus Salam Khan

**Affiliations:** 1 Emergency Medicine, Shifa International Hospital, Islamabad, PAK; 2 Pathology, Poonch Medical College, Rawalakot, PAK; 3 Emergency Medicine, Medical University of Lodz, Lodz, POL

**Keywords:** bicarbonate, metabolic acidosis, sodium bicarbonate

## Abstract

The management of acid-base disorders always calls for precise diagnosis and treatment of the underlying disease. Sometimes additional means are necessary to combat systemic acidity itself. In this systematic review, we discuss the concept and some specific aspects of bicarbonate therapy for critically ill patients with metabolic acidosis (i.e., patients with blood pH < 7.35).

We conducted a systematic literature review of three online databases (PubMed, Google Scholar, and Cochrane) in November 2018 to validate usage of bicarbonate therapy for critically ill patients with metabolic acidosis. Twelve trials and case series were included in the final analysis, from which we assessed population, intervention, comparison, and outcome data.

The current literature suggests limited benefit from bicarbonate therapy for patients with severe metabolic acidosis (pH < 7.1 and bicarbonate < 6 mEq/L). However, bicarbonate therapy does yield improvement in survival for patients with accompanying acute kidney injury.

## Introduction and background

Metabolic acidosis is defined as low blood pH levels (pH < 7.35) due to a reduced concentration of bicarbonate (HCO3-) in the serum with a secondary reduction in arterial pressure of carbon dioxide (PaCO2) [1-2]. It is frequently encountered among patients hospitalised in intensive care units (ICU) with the incidence of 8% to 64% [1-3].

Blood gas analysis often consists of three parameters: total concentration of carbon dioxide in the blood, plasma partial pressure of carbon dioxide (pCO2), and plasma HCO3- concentration. The last parameter is usually obtained based on pH and pCO2 described by the Henderson-Hasselbalch equation [4-6]. Therapy with sodium bicarbonate is indicated for disorders associated with the loss of HCO3- (e.g., diarrhoea, renal tubular acidosis), but the efficacy of sodium bicarbonate therapy to correct metabolic acidosis caused by other reasons has not been established and is the subject of ongoing research [4,7-9].

The management of acid-base disorders always calls for precise diagnosis and treatment of the underlying disease. Sometimes it requires additional means to combat abnormal systemic acidity. In this systematic review, we review the concept and some specific aspects of bicarbonate therapy for critically ill patients with metabolic acidosis.

## Review

Material and methods

**Figure 1:**
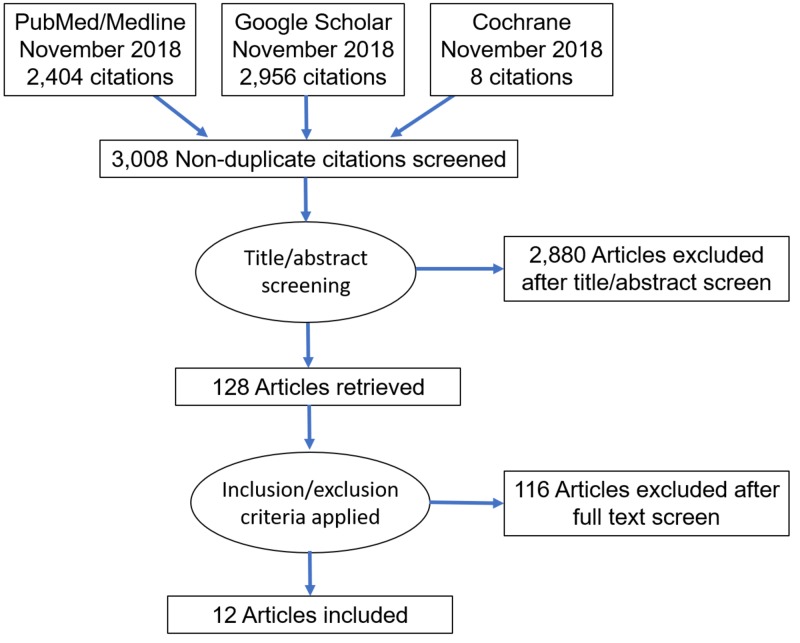
PRISMA flow diagram PRISMA, Preferred Reporting Items for Systematic Reviews and Meta-analyses.

Two authors individually performed a systematic literature review of three online databases (PubMed/MEDLINE, Google Scholar, and Cochrane) till November 2018 with the following search terms: "bicarbonate" OR "bicarbonate therapy" AND "metabolic acidosis" OR "lactic acidosis" OR “ketoacidosis” OR “intensive care unit”. Inclusion criteria were (i) reporting on bicarbonate usage in metabolic acidemia, (ii) article in English. Exclusion criteria were (i) conference abstract, reports and similar (ii) participants younger than 18 years. After the search, 3,008 articles were screened by title and abstract. Of those, 128 relevant articles underwent a detailed review of relevance for full-text. The disagreements were resolved by mutual discussion (Figure 1).

During the research, we identified 12 articles on bicarbonate therapy for critically ill patients with metabolic acidosis. To identify other relevant studies, we manually scanned reference lists from the identified trials and review articles. Our review follows the guidelines set by the Preferred Reporting Items for Systematic Reviews and Meta-analyses (PRISMA) statement [10].

Results

Twelve trials and case series were included in the final analysis. We extracted population, intervention, comparison, and outcome (PICO) data from the 12 included articles. Summaries of the relevant studies are presented in Table 1. 

**Table 1:** PICO data from included studies AKI, acute kidney infection; BUN, blood urea nitrogen; CI, confidence interval; CO, cardiac output; CPR, cardiopulmonary resuscitation; DPG, 2,3-diphosphoglycerate; HR, heart rate; ICU, intensive care unit; IV, intravenous; MAP, mean arterial pressure; PaCO2, partial pressure of carbon dioxide in arterial blood; PICO, population, intervention, comparison, outcome; PCO2, partial pressure of carbon dioxide; Pt, patients; ROSC, return of spontaneous circulation; SB, sodium bicarbonate; SD, standard deviation.

Study, year	Population	Intervention	Comparison	Outcome	Conclusions/Comments
Jung et al., 2011 [3]	155 pt in ICU with severe acidemia (pH < 7.2)	57 pt received bicarbonate therapy	Length of vasopressor treatment, Length of mechanical ventilation, ICU length of stay, Mortality in the ICU	No significant differences	Sodium bicarbonate does not influence outcomes of severe acidemia
Cooper et al., 1990 [11]	14 pt with metabolic acidosis (bicarbonate < 17 mmol/L and base excess <10) and increased arterial lactate (mean, 7.8 mmol/L)	SB (2 mmol/kg body weight over 15 minutes) / sodium chloride	arterial pH and partial pressure of CO_2_, serum bicarbonate, plasma ionized calcium, pulmonary capillary wedge pressure, cardiac output, mean arterial pressure, hemodynamic responses	SB increased arterial pH (7.22 to 7.36, p < 0.001), serum bicarbonate (12 to 18 mmol/L, P < 0.001), and partial pressure of CO_2_ in arterial blood (PaCO_2_) (35 to 40 mm Hg, P < 0.001) and decreased plasma ionized calcium (0.95 to 0.87 mmol/L, P < 0.001). SB and sodium chloride both transiently increased pulmonary capillary wedge pressure (15 to 17 mm Hg, and 14 to 17 mm Hg, P < 0.001) and cardiac output (18% and 16%, P< 0.01). The mean arterial pressure and hemodynamic responses was unchanged.	Correction of acidaemia using SB does not improve hemodynamic in critically ill pt
Mathieu et al.,1991 [12]	10 pt with metabolic acidosis, increased arterial plasma lactate concentrations (greater than 2.45 mmol/L), and no severe renal failure (creatinine < 250 mmol/L [less < 2.3 mg/dL])	SB and sodium chloride in randomized order.	Arterial and venous blood gas measurements, plasma electrolytes (sodium, potassium, chloride), osmolality and lactate, DPG, and oxygen hemoglobin affinity, hemodynamic variables, oxygen delivery, oxygen consumption measurements	SB administration increased arterial and venous pH, serum bicarbonate, and the partial pressure of CO_2_in arterial and venous blood. No other significant differences.	Administration of SB did not improve hemodynamic variables in pt with lactic acidosis, but did not worsen tissue oxygenation
Stacpoole et al., 1994 [13]	126 pt with lactic acidosis, defined as arterial blood lactate greater than or equal to 5 mmol/L and either arterial pH ≤ 7.35 or base deficit > 6 mmol/L.	Placebo vs dichloroacetate as specific lactate-lowering therapy. 44 pt (35%) received at least 50 mmol of IV SB within the first 24 hours of entry	Hemodynamics, mortality	In pt receiving SB, neither acid-base nor hemodynamic status improved.	
Fang et al., 2008 [14]	94 pt with sepsis and hypotension	Injections within 15 min at initial treatment. 32 received 5 ml/kg normal saline; 30 received 5 ml/kg 3.5% sodium chloride, 32 received 5 ml/kg 5% SB	Cardiac output, systolic blood pressure, mean arterial pressure, body temperature, heart rate, respiratory rate, blood gases, mortality rate after 28 days	No differences among the three groups in outcome measures. Improvement of MAP and CO started earlier in the SB group than in the normal saline and sodium chloride groups. SB increased the base excess but did not alter blood pH, lactic acid or bicarbonatevalues	SB confers a limited benefit
El-Solh et al., 2010 [15]	72 pt: 36 pt with septic shock and elevated blood lactate levels, 36 pt-matched controls	Continuous infusion of bicarbonate therapy	Time until reversal of shock, time to liberation of mechanical ventilation, length of intensive care unit, 28-day mortality	Bicarbonate group: median time to liberation of mechanical ventilation was reduced (10 days [95% CI, 5.0 to 13.0] vs. 14 days [95% CI, 9.0 to 19.0], p = 0.02) and the length of intensive care unit stay was shorter (11.5 days (95% CI, 6.0 to 16.0) vs. 16.0 days (95% CI, 13.5 to 19.0), p = 0.01). No difference in time until reversal of shock and 28-day mortality.	Infusion of SB in septic pt with arterial hyperlactatemia may facilitate weaning from mechanical ventilation and reduce length of ICU stay
Ahn et al., 2018 [16]	50 pt with >10 min CPR and with severe metabolic acidosis(pH<7.1 or bicarbonate < 10 mEq/L)	Sodium bicarbonate(n=25) or normal saline (n=25)	Return of spontaneous circulation, change of acidosis, good neurologic survival.	Sodium bicarbonate group had significant effect on pH (6.99 vs.6.90, P = 0.038) and bicarbonate levels (21.0 vs. 8.0 mEq/L, P = 0.007). However, no significant differences showed between sodium bicarbonate and placebo groups in sustained ROSC (4.0% vs. 16.0%, P = 0.349) or good neurologic survival at one month (0.0% vs.4.0%, P = 1.000)	The use of sodium bicarbonate improved acid-base status, but did not improve the rate of ROSC and good neurologic survival
Jaber et al., 2018 [17]	389 pt with severe acidaemia (pH ≤ 7.20, PaCO_2_ ≤45 mm Hg, SB concentration ≤ 20 mmol/L), arterial lactate concentration of at least 2 mmol/L)	194 in the control group, 195 in the SB group (125–250 mL 4.2% SB IV infusion in 30 min to obtain pH > 7.30)	Survival at 28 days, organ failure at seven days.	For survival (46% [95% CI 40–54] vs 55% [49–63]; p = 0.09 for organ failure absolute difference estimate –5.5%, 95% CI, –15.2 to 4.2; p = 0.24	No effect on the primary composite outcome. Improvement in AKI subgroup.
Zhang et al., 2018 [18]	1718 septic pt with metabolic acidosis (pH < 7.3), 500 pairs of pt formed	500 pt received bicarbonate therapy	Survival	No significant mortality effect in the overall population (HR, 1.04; 95% CI 0.86 to 1.26; p = 0.67), bicarbonate therapy beneficial in pt with AKI stage 2 or 3	SB infusion was not associated with improved outcome in septic pt with metabolic acidosis, but it was associated with improved survival in septic pt with AKI stage 2 or 3 and severe acidosis
Kim et al., 2013 [19]	103 pt with lactic acidosis	69 pt received bicarbonate therapy	Survival	SB administration (p = 0.016) was associated with higher mortality.	Sodium bicarbonate should be prescribed with caution in the case of lactic acidosis because SB administration may affect mortality
Mintzer et al., 2015 [20]	12 neonates (500 to 1250 g)	SB 'half' corrections (0.3 * Weight (kg) * Base Deficit [mmol l(-1)]) for presumed renal losses	Regional oxygen saturation, fractional tissue oxygen extraction	SB corrections lowered base deficit from 7.6 ± 1.8 to 3.4 ± 2.1 mmol l(-1) (P < 0.05), and increased median (±SD) pH from 7.23 ± 0.06 to 7.31 ± 0.05 (P < 0.05). No significant changes in blood pressure, pulse oximetry, PCO2, lactate, sodium, BUN, creatinine, hematocrit Cerebral/renal/splanchnic regional oxygen saturation, fractional tissue oxygen extraction were observed.	Further prospective evaluation to differentiate metabolic acidosis due to oxygen delivery/consumption imbalance versus renal bicarbonate losses.
Lee et al., 2015 [21]	109 pt with severe sepsis, pt with lactic acidosis	All pt received SB	seven-day mortality rate	The seven-day mortality rate was 71.6%	Descriptive

Discussion

Metabolic acidosis is an acid-base disorder characterised by low serum pH from reduced HCO3- levels following a compensatory decrease in PaCO2 [1-2]. When blood pH is < 7.20, acidosis is severe [1-2]. There are two main mechanisms underlying metabolic acidosis: a deficit in HCO3- (loss by kidneys or gastrointestinal system) or addition of strong acids, where lactic acidosis and ketoacidosis are the two most common causes of severe metabolic acidosis [2,22-23].

Capnography is the primary diagnostic method of metabolic acidosis in spontaneously breathing patients referred to the emergency wards. However, arterial blood gas is the gold standard tool for diagnosis, the results of which guide the treatment [24]. Metabolic acidosis affects the cardiovascular, respiratory, metabolic, cerebral, renal, haematological, endocrine, musculoskeletal, and immunological systems [25-29].

Bicarbonate Therapy

Buffers are substances that counteract changes in pH [9], and sodium bicarbonate is the most frequently used buffer [30-31]. The main reason to commence sodium bicarbonate therapy is to prevent or reverse the effects of metabolic acidemia, especially in the cardiovascular system [25]. For bicarbonate therapy to be effective, plasma HCO3- levels must be increased to 8 mmol/L to 10 mmol/L. There are no guidelines stating exactly how to achieve these levels given a variety of influencing factors (e.g., vomiting, renal failure) [25].

When a patient is given bicarbonate, the production of lactate is stimulated in lactic acidosis [32-34] diabetic ketoacidosis [35], and hemorrhagic shock [36]. Sodium bicarbonate should be dispensed as an infusion over several hours. In cases of severe acidemia, a bolus may be considered. The clinical effect can be assessed at least 30 minutes after infusion [25].

Complications of Bicarbonate Therapy

Sodium bicarbonate infusions may result in hypernatremia and hyperosmolality. However, the addition of sodium chloride and 5% dextrose creates an isotonic solution and will help prevent these adverse effects [25]. Extracellular-fluid volume overload is another negative consequence of bicarbonate therapy, and the risk is higher among patients with congestive heart failure and/or renal failure. To prevent extracellular-fluid volume overload, loop diuretics (e.g., furosemide) should be used. In worst-case scenarios, hemofiltration and/or dialysis may be needed [25].

In cases of lactic acidosis or ketoacidosis, the simulation of 6-phosphofructokinase activity and organic acid production should be considered, as the overproduction of organic acid may limit the effects of alkalizing agents [25].

Bicarbonate Therapy for Patients with Metabolic Acidosis

Three recent studies on 150 patients with metabolic acidemia (pH ≤ 7.35) and increased lactate concentrations (serum lactate > 2.45 or 5 mmol/L) failed to prove sodium bicarbonate offered a limited benefit on mortality and hemodynamic variables [11-13]. In another study, Fang et al. evaluated a cohort of 94 patients with sepsis assigned into three groups receiving 5 mL/kg normal saline, 5 mL/kg 3.5% sodium chloride, and 5 mL/kg 5% sodium bicarbonate. They reported no differences in cardiac output, mean arterial pressure heart rate or respiratory rate eight hours following infusion, and no significant differences were observed in mortality rate after 28 days. However, patients receiving sodium bicarbonate showed improved hemodynamic parameters earlier than those in other groups [14].

Kraut et al. surveyed nephrologists and critical care physicians on their use of bases in treating acute severe organic acidosis [37]. While results varied among individual physicians from both specialties, a larger percentage of nephrologists recommended administration of base for the treatment of lactic acidosis and ketoacidosis than critical care physicians (lactic acidosis, 86% vs.67%; ketoacidosis, 60% vs.28%). Sodium bicarbonate was the most utilized form of base used for treatment (> 75%) [37].

The first positive study on the benefits of sodium bicarbonate therapy was published in 2010 by El-Solh et al. [17]. They compared 36 patients with septic shock and elevated lactate levels with 36 controls with septic shock match-paired by age, site of infection, and mortality prediction based on the Acute Physiology and Chronic Health Evaluation II (APACHE II) scale. Bicarbonate infusion (0.15 M, 0.1 to 0.2 mmol/kg ideal body weight/hour) was initiated in patients with increased arterial lactate levels, and pH < 7.3 and was stopped when the pH reached 7.35 to 7.4. The therapy did not reduce the time of shock reversal. Nevertheless, bicarbonate infusion shortened the time of mechanical ventilation (10 days [95% confidence interval (CI), 5.0 to 13.0] vs. 14 days [95% CI, 9.0 to 19.0], p = 0.02) and duration of ICU stay (11.5 days [95% CI, 6.0 to 16.0) vs. 16.0 days [95% CI, 13.5 to 19.0), p = 0.01) [15].

In 2013, Chen et al. published results of their prospective randomized, double-blind, controlled clinical trial involving 65 patients with hypoperfusion-induced lactic acidemia due to septic shock. They compared early the efficacy of sodium bicarbonate therapy between two groups. In the first group of 35 patients, sodium bicarbonate was given in stages. In the first stage, it was administered via intravenous (IV) drip until blood pH reached at least 7.15. In the second stage, sodium bicarbonate was given by IV drip until blood pH7 reached at least 7.25 after six hours. In the other group of 30 patients, the drug was given via IV until the blood pH reached 7.15. The staging group had a lower incidence of organ dysfunction, shorter time of mechanical ventilation, lower maximum sequential organ failure assessment (SOFA) score, lower change in SOFA score, shorter duration of ICU and hospital stays, and decreased mortality compared to the control group [38].

Studies published in 2018 yielded further insights into bicarbonate therapy. Ahn et al. conducted a prospective, double-blind, randomized placebo-controlled pilot trial of 50 patients who could not achieve a return of spontaneous circulation (ROSC) after 10 minutes of cardiopulmonary resuscitation and with severe metabolic acidosis (pH < 7.1 or HCO3- < 10 mEq/L). Ahn et al. reported improved acid-base status, but no change is the rate of ROSC and good neurologic survival for the patients receiving sodium bicarbonate (50 mEq/L) [16]. In June 2018, a multicenter, open-label, randomized controlled, phase III trial conducted in 26 intensive care units in France was published. From May 2015 to May 2017, 389 patients with severe acidemia (pH ≤ 7.20) were enrolled into the intention-to-treat analysis (194 in the control group and 195 in the bicarbonate group, who received 4.2% natrium bicarbonate infusion to raise the pH level to at least 7.3). Any organ failure within seven days occurred in 138 (71%) of 194 patients in the control group and 128 (66%) of 195 in the treatment group (absolute difference estimate, -5.5%; 95% CI, -15.2 to 4.2; p = 0.24). No significant difference was observed for 28-day survival (46% [95% CI, 40 to 54] vs 55% [95% CI, 49 to 63] respectively, p = 0.09). However, survival by day 28 was significant for a subgroup of patients with acute kidney injury (63% [95% CI, 52 to 72] for bicarbonate therapy vs.46% [95% CI, 35 to 55]; p = 0·0283 for controls). Additionally, the number of days free from renal-replacement therapy and vasopressors was higher. These findings suggest that unlike the overall population of patients with metabolic acidosis, those suffering from concomitant acute kidney injury may experience improved outcomes and a reduced rate of mortality from enrolment to day 28 with sodium bicarbonate infusion therapy [19]. Similarly, Zhang et al. studied 1718 septic patients (1218 controls and 500 patients who received sodium bicarbonate) and reported no significant mortality change in the overall population (hazard ratio [HR], 1.04; 95% CI, 0.86 to 1.26; p = 0.67], but bicarbonate proved to be beneficial in patients with acute kidney injury (HR, 0.74; 95% CI, 0.51 to 0.86; p = 0.021) [20].

Limitations

Our review had several limitations. Data were only searched in three databases, and the inclusion of other databases could increase the range of articles found. In addition, we limited our inclusion to studies published in English. Given our focus was gathering information regarding bicarbonate therapy, we did not evaluate the methodologic quality of the included studies. These limitations did not substantially alter the results. A meta-analysis was not conducted given the heterogeneity of the data.

## Conclusions

The current literature suggests bicarbonate therapy offers limited benefits as a treatment of patients with severe metabolic acidosis (pH < 7.1 and HCO3- < 6 mEq/L) and patients with accompanying acute kidney injury. Further studies assessing treatments may be of interest in the population of patients with metabolic acidosis in the ICU. Details on the entering and exiting point of therapy should be evaluated as well as a base solution with dosage. Sodium bicarbonate therapy can offer effective outcomes in appropriate, carefully selected patients.

## References

[1] Kraut JA, Madias NE (2010). Metabolic acidosis: pathophysiology, diagnosis and management. Nat Rev Nephrol.

[2] Ellis MF (2015). Use of bicarbonate in patients with metabolic acidosis. Crit Care Nurse.

[3] Jung B, Rimmele T, Le Goff C (2011). Severe metabolic or mixed acidemia on intensive care unit admission: incidence, prognosis andadministration of buffer therapy. A prospective, multiple-center study. Crit Care.

[4] Adeva-Andany MM, Fernández-Fernández C, Mouriño-Bayolo D, Castro-Quintela E, Domínguez-Montero A (2014). Sodium bicarbonate therapy in patients with metabolic acidosis. Sci World J.

[5] Aiken CGA (2013). History of medical understanding and misunderstanding of acid-base balance. J Clin Diagn Res.

[6] Berend K (2013). Acid-base pathophysiology after 130 years: confusing, irrational and controversial. J Nephrol.

[7] Ammari AN, Schulze KF (2002). Uses and abuses of sodium bicarbonate in the neonatal intensive care unit. Curr Opin Pediatr.

[8] Forsythe SM, Schmidt GA (2000). Sodium bicarbonate for the treatment of lactic acidosis. Chest.

[9] Gehlbach BK, Schmidt GA (2004). Bench-to-bedside review: treating acid-base abnormalities in the intensive care unit - the role of buffers. Crit Care.

[10] Hutton B, Salanti G, Caldwell DM (2015). The PRISMA extension statement for reporting of systematic reviews incorporating network meta-analyses of health care interventions: checklist and explanations. Ann Intern Med.

[11] Cooper DJ, Walley KR, Wiggs BR, Russell JA (1990). Bicarbonate does not improve hemodynamics in critically ill patients who have lactic acidosis. A prospective, controlled clinical study. Ann Intern Med.

[12] Mathieu D, Neviere R, Billard V, Fleyfel M, Wattel F (1991). Effects of bicarbonate therapy on hemodynamics and tissue oxygenation in patients with lactic acidosis: a prospective, controlled clinical study. Crit Care Med.

[13] Stacpoole PW, Wright EC, Baumgartner TG (1994). Natural history and course of acquired lactic acidosis in adults. Am J Med.

[14] Fang ZX, Li YF, Zhou XQ (2008). Effects of resuscitation with crystalloid fluids on cardiac function in patients with severe sepsis. BMC Infect Dis.

[15] El-Solh AA, Abou Jaoude P, Porhomayon J (2010). Bicarbonate therapy in the treatment of septic shock: a second look. Intern Emerg Med.

[16] Ahn S, Kim Y-J, Sohn CH, Seo DW, Lim KS, Donnino MW, Kim WY (2018). Sodium bicarbonate on severe metabolic acidosis during prolonged cardiopulmonary resuscitation: a double-blind, randomized, placebo-controlled pilot study. J Thorac Dis.

[17] Jaber S, Paugam C, Futier E (2018). Sodium bicarbonate therapy for patients with severe metabolic acidaemia in the intensive care unit (BICAR-ICU): a multicentre, open-label, randomised controlled, phase 3 trial. Lancet.

[18] Zhang Z, Zhu C, Mo L, Hong Y (2018). Effectiveness of sodium bicarbonate infusion on mortality in septic patients with metabolic acidosis. Intensive Care Med.

[19] Kim HJ, Son YK, An WS (2013). Effect of sodium bicarbonate administration on mortality in patients with lactic acidosis: a retrospective analysis. PLoS One.

[20] Mintzer JP, Parvez B, Alpan G, LaGamma EF (2015). Effects of sodium bicarbonate correction of metabolic acidosis on regional tissue oxygenation in very low birth weight neonates. J Perinatol.

[21] Lee SM, Kim SE, Kim E Bin, Jeong HJ, Son YK, An WS (2015). Lactate clearance and vasopressor seem to be predictors for mortality in severe sepsis patients with lactic acidosis supplementing sodium bicarbonate: a retrospective analysis. PLoS One.

[22] Gabow PA, Kaehny WD, Fennessey PV, Goodman SI, Gross PA, Schrier RW (1980). Diagnostic importance of an increased serum anion gap. N Engl J Med.

[23] Sabatini S, Kurtzman Kurtzman, NA NA (2009). Bicarbonate therapy in severe metabolic acidosis. J Am Soc Nephrol.

[24] Taghizadieh A, Pouraghaei M, Moharamzadeh P, Ala A, Rahmani F, Basiri Sofiani K (2016). Comparison of end-tidal carbon dioxide and arterial blood bicarbonate levels in patients with metabolic acidosis referred to emergency medicine. J Cardiovasc Thorac Res.

[25] Adrogué HJ, Madias NE (1998). Management of life-threatening acid-base disorders. N Engl J Med.

[26] Patel MP, Ahmed A, Gunapalan T, Hesselbacher SE (2018). Use of sodium bicarbonate and blood gas monitoring in diabetic ketoacidosis: a review. World J Diabetes.

[27] Kimmoun A, Novy E, Auchet T, Ducrocq N, Levy B (2015). Hemodynamic consequences of severe lactic acidosis in shock states: from bench to bedside. Crit Care.

[28] Kraut JA, Madias NE (2012). Treatment of acute metabolic acidosis: a pathophysiologic approach. Nat Rev Nephrol.

[29] Cooper DJ, Worthley LI (1987). Adverse haemodynamiceffects of sodium bicarbonate in metabolic acidosis. Intensive Care Med.

[30] Ahmed AR, Lappin D (2018). Oral alkali therapy and the management of metabolic acidosis of chronic kidney disease: a narrative literature review. World J Nephrol.

[31] Kovesdy CP, Kalantar-Zadeh K (2010). Oral bicarbonate: renoprotective in CKD?. Nat Rev Nephrol.

[32] Rhee KH, Toro LO, McDonald GG, Nunnally RL, Levin DL (1993). Carbicarb, sodium bicarbonate, and sodium chloride in hypoxic lactic acidosis: effect on arterial blood gases, lactate concentrations, hemodynamic variables, and myocardial intracellular pH. Chest.

[33] Arieff AI, Leach W, Park R, Lazarowitz VC (1982). Systemic effects of NaHCO3 in experimental lactic acidosis in dogs. Am J Physiol.

[34] Graf H, Leach W, Arieff AI (1985). Metabolic effects of sodium bicarbonate in hypoxic lactic acidosis in dogs. Am J Physiol.

[35] Bureau MA, Bégin R, Berthiaume Y, Shapcott D, Khoury K, Gagnon N (1980). Cerebral hypoxia from bicarbonate infusion in diabetic acidosis. J Pediatr.

[36] Beech JS, Williams SC, Iles RA, Cohen RD, Nolan KM, Evans SJ, Going TC (1995). Haemodynamic and metabolic effects in diabetic ketoacidosis in rats of treatment with sodium bicarbonate or a mixture of sodium bicarbonate and sodium carbonate. Diabetologia.

[37] Kraut JA, Kurtz I (2006). Use of base in the treatment of acute severe organic acidosis by nephrologists and critical care physicians: results of an online survey. Clin Exp Nephrol.

[38] Chen XF, Ye JL, Zhu ZY (2013). The use of sodium bicarbonate in stages in treating hypoperfusion induced lactic acidemia in septic shock [Article in Chinese]. Zhonghua Wei Zhong Bing Ji Jiu Yi Xue.

